# The Incidence of Misdiagnosis in Patients with Ehlers–Danlos Syndrome

**DOI:** 10.3390/children12060698

**Published:** 2025-05-29

**Authors:** Chanbin Lee, Pradeep Chopra

**Affiliations:** 1 Warren Alpert Medical School, Brown University, Providence, RI 02903, USA; chanbin_lee@brown.edu; 2 Center for Complex Conditions, Pawtucket, RI 02860, USA

**Keywords:** Ehlers–Danlos Syndrome, chronic pain, misdiagnosis

## Abstract

**Background**: Misdiagnosis, defined as the incorrect identification of a condition or the failure to identify a condition altogether, can lead to delayed treatment, unnecessary interventions, and avoidable morbidity and mortality. Ehlers–Danlos Syndrome (EDS) is a complex pain disorder that is often misdiagnosed or underdiagnosed due to lack of awareness among healthcare providers and variability in diagnostic criteria. **Objectives**: This study aimed to determine the misdiagnosis rate of hypermobile EDS (hEDS) with psychiatric disorders by physicians who are not board-certified in psychiatry. **Methods**: Between January 2010 and December 2018, the medical records of 429 patients who were diagnosed with hEDS were reviewed and analyzed. During the process of taking a history, patients were asked if they had previously been told by physicians who were not board-certified in psychiatry that their symptoms were “in their head”, that they were “making it up” or seeking attention, or that they might suffer from Munchausen syndrome by proxy or a factitious disorder, or if such physicians had diagnosed them with conversion disorder. The Brown University Human Research Protection Program determined that the proposed activity was not research involving human subjects. **Results**: A retrospective chart review was conducted. Among the 429 patients, 405 patients (94.4%) said yes to at least one of the questions, with only 24 patients (5.6%) not having been misdiagnosed with psychiatric illnesses. A total of 378 patients (88%) were told that they were “making it up”, 326 patients (76%) were told that they were attention-seeking, 286 patients (67%) were diagnosed with conversion disorder, 255 patients (60%) were told that “it was in their head”, and 16 patients (4%) were diagnosed with Munchausen syndrome by proxy or a factitious disorder. **Conclusions**: Misdiagnosis of Ehlers–Danlos Syndrome is a pervasive issue with profound implications for patients’ physical, mental, and economic well-being. By addressing the underlying causes of misdiagnosis and implementing strategies for improved recognition, the healthcare system can significantly enhance outcomes for individuals who are affected by these complex disorders.

## 1. Introduction

Misdiagnosis remains one of the most pervasive challenges in modern medicine, with far-reaching implications for patients’ health and emotional well-being, as well as healthcare systems. Defined as the incorrect identification of a condition or the failure to identify a condition altogether, misdiagnosis can lead to delayed treatment, unnecessary interventions, and avoidable morbidity and mortality. The prevalence of misdiagnosis is alarming across various medical specialties, with studies estimating that 10% to 15% of all medical diagnoses are incorrect, and that nearly every individual will experience a diagnostic error during their lifetime [1,2,3,4,5].

Ehlers–Danlos Syndromes (EDSs) comprise a group of heritable connective tissue disorders that can affect the skin, ligaments, joints, and blood vessels. Multisystemic symptoms in patients with EDS typically include skin hyperextensibility, joint hypermobility and instability, and vascular fragility. The *2017 International Classification for the Ehlers-Danlos Syndromes* recognizes 13 subtypes: arthrochalasia, brittle cornea syndrome, cardiac–vascular EDS, classical EDS, classic-like EDS, dermatosparaxis, hypermobile EDS, kyphoscoliotic EDS, musculocontractural EDS, myopathic EDS, periodontal EDS, spondylodysplastic EDS, and vascular EDS [6]. Among the 13 subtypes of EDS, causative genes for 12 subtypes have been identified. The gene for hypermobile EDS (hEDS), which is the most common type of EDS, has not been identified [6,7,8,9].

With no specific causative gene being identified to date, the diagnostic criteria for hEDS remain clinical [9,10,11]. A diagnosis of hEDS requires fulfillment of criteria 1, 2, and 3. Criterion 1 investigates general hypermobility using a Beighton score, a nine-point scoring system based on the physical exam findings of the present or the past on joint mobility [6,12,13]. Criterion 2 investigates family history and a comprehensive physical exam that focuses on the cardiac, dermatological, dental, vascular, and musculoskeletal systems. Joint instability and pain are also investigated in this criterion [6,10]. Criterion 3 excludes any other pathologies that have overlapping symptoms with hEDS [6,9,10,11]. When patients experience general hypermobility but do not meet all three criteria, they are diagnosed with hypermobility spectrum disorders [8].

Multiple studies have shown that hEDS patients are often under- and misdiagnosed [14,15,16]. Chronic pain, although not a required criteria for hEDS diagnosis, is extremely common in patients with hEDS [6,17]. When children with hEDS are correctly diagnosed, they can be explained that their psychological symptoms are secondary to an organic cause. However, when these patients with hEDS are misdiagnosed with primary psychological disorders that they do not have, they undergo inappropriate psychiatric interventions, while the underlying medical condition continues to progress untreated. Studies have found that the misdiagnosis of EDS leads to increased risks of decreased physical activity in school and developing autism or ADHD [17,18,19].

Additionally, misdiagnosed children may develop mistrust toward medical professionals, complicating future healthcare interactions. The stigma associated with psychological diagnoses, when patients do, indeed, have hEDS, can exacerbate emotional distress, especially if peers, educators, or family members misinterpret the child’s symptoms as behavioral issues or attention-seeking rather than legitimate medical concerns [17,20].

This article aims to determine the misdiagnosis rate of EDS as psychiatric disorders by physicians who are not board-certified psychiatrists, explore contributing factors to the high misdiagnosis rate, and identify approaches to reduce diagnostic errors in patients with EDS.

## 2. Materials and Methods

The medical records of 429 patients who were diagnosed with hypermobile Ehlers–Danlos Syndrome (hEDS) and treated at the Center for Complex Conditions, Pawtucket, RI, USA, between January 2010 and December 2018 were retrospectively reviewed and analyzed using Microsoft^®^ Excel Version 16.89.1 (24091630). As part of routine intake procedures, patients completed questionnaires regarding their diagnostic experiences, specifically if their symptoms had ever been labeled as being “in their head” or if they had been accused of fabricating symptoms (written as “making it up” in the form) or being attention-seeking, diagnosed with Munchausen syndrome by proxy or a factitious disorder, or diagnosed with conversion disorder (Table 1). The intake questionnaires listed in Table 1 were developed from the list of accusations that patients with hEDS or other chronic pain conditions repeatedly reported during clinical examinations. They are not based on standardized instruments, as such validated tools, developed for complex chronic pain conditions, are lacking. The definitions of conversion disorder, Munchausen by proxy, and Munchausen were described to the patients, and the term “making it up” was used instead of fabricating symptoms in consideration of the medical literacy of the patients [21].

Patients who responded positively to at least one question in Table 1 were further queried regarding whether these diagnoses were made by board-certified psychiatrists during their evaluations. The criteria for misdiagnosis included pain being attributed to primary psychiatric illness diagnoses made by non-psychiatry board-certified physicians for patients who were later diagnosed with hEDS, which is a clinical diagnosis mainly based on a physical exam. Some patients had subsequent evaluations by board-certified psychiatrists confirming the absence of such psychiatric misdiagnoses, but the self-reported absence of it did not lead to exclusion in the study population. Patients who were both diagnosed with hEDS and any other psychiatric disorders were considered not to be misdiagnosed. Patients whose pain was attributed to psychiatric illnesses that were secondary to EDS or other organic causes were categorized correctly diagnosed. The Brown University Human Research Protection Program determined that the proposed activity was not research involving human subjects. Generative artificial intelligence (GenAI) was not used in this paper.

## 3. Results

### Misdiagnosis of Patients with hEDS with Psychiatric Illnesses by Non-Psychiatry Board-Certified Physicians

**Figure 1:**
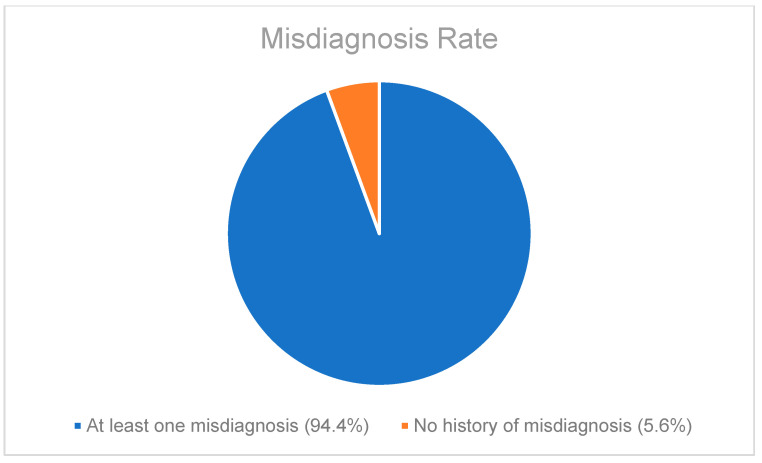
Pie chart illustrating the percentage of patients who had been misdiagnosed as having psychiatric illnesses with at least one of the five misdiagnosis categories included during routine history taking.

**Figure 2:**
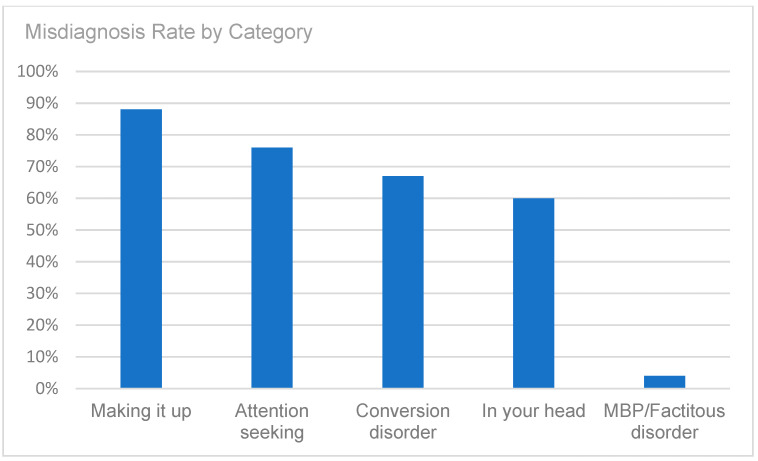
Misdiagnosis rate by category (symptoms were “in their head”, patients were “making it up”, patients were seeking attention, diagnosis of Munchausen syndrome by proxy or factitious disorder, or diagnosis of conversion disorder).

**Figure 3:**
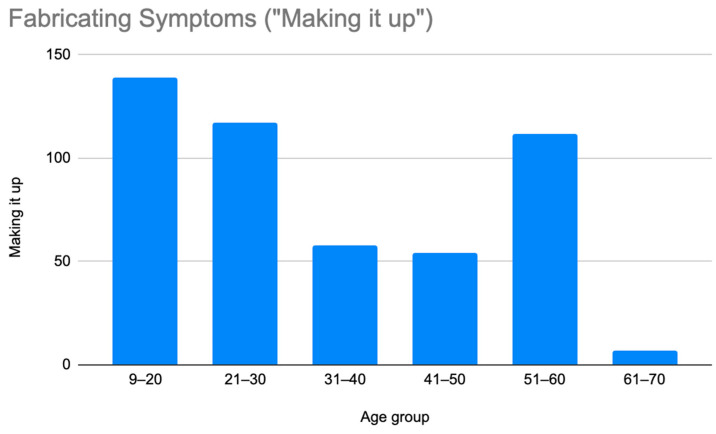
Prevalence of patients who were misdiagnosed by claims that they were making their symptoms up by age group.

**Figure 4:**
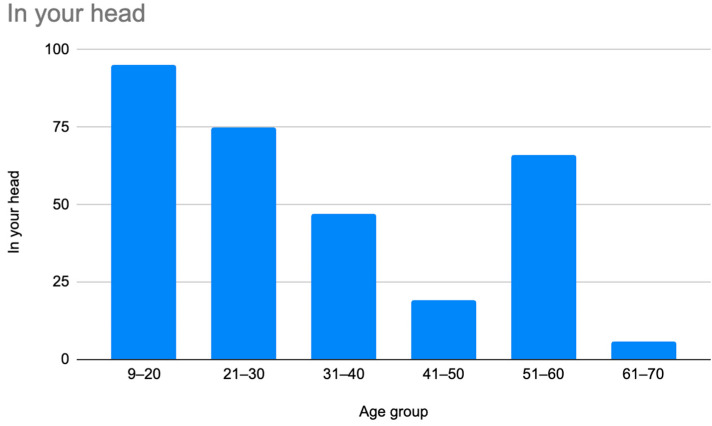
Prevalence of patients who were misdiagnosed by claims that their symptoms were “in their head” by age group.

**Figure 5:**
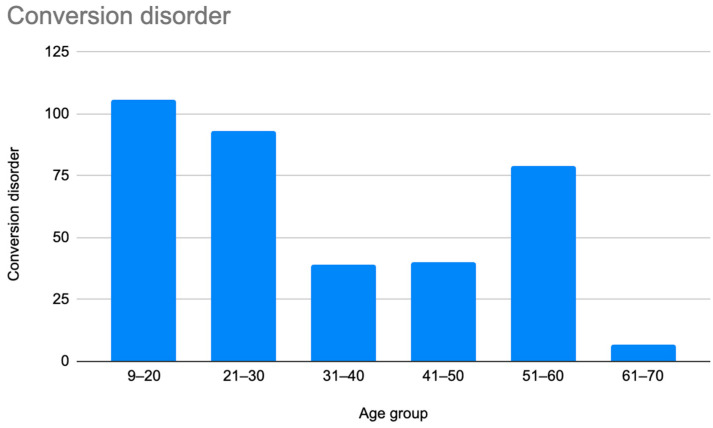
Prevalence of patients who were misdiagnosed with conversion disorder by age group.

**Figure 6:**
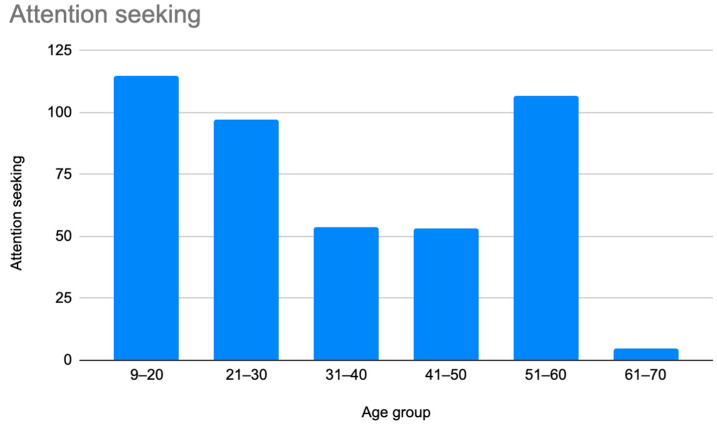
Prevalence of patients who were misdiagnosed as attention-seeking by age group.

**Figure 7:**
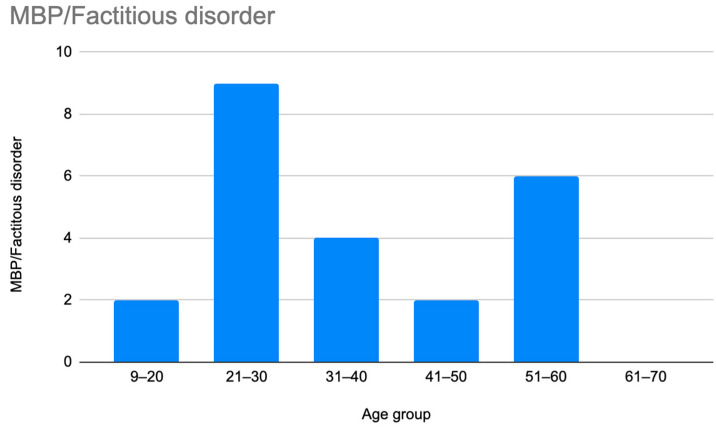
Prevalence of patients who were accused of having Munchausen syndrome by proxy or Munchausen syndrome by age group.

**Figure 8:**
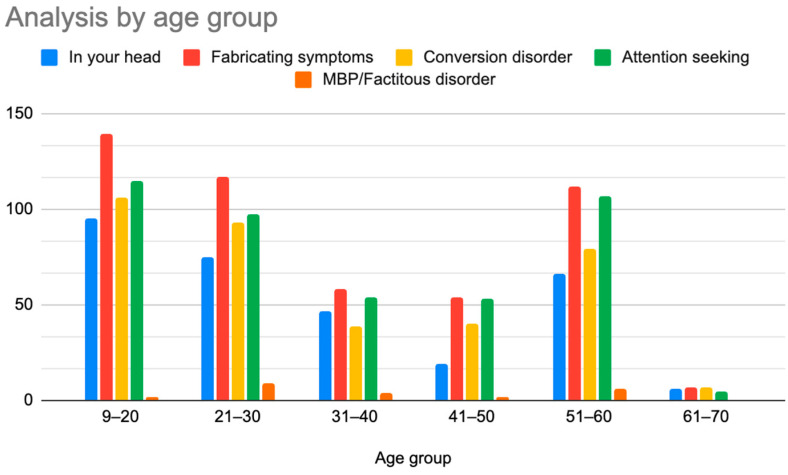
Prevalence of patients who were diagnosed with primary psychiatric diagnoses by age group.

A retrospective chart review revealed that among the 429 patients who were clinically diagnosed with hEDS (94.8% female, mean age 27.9 ± 13.2 years), 405 patients (94.4%) had experienced misdiagnosis in at least one of the five evaluated categories (symptoms were “in their head”, “making it up”, seeking attention, Munchausen syndrome by proxy or a factitious disorder, or conversion disorder), as shown in Figure 1. Only 24 patients (5.6%) had not received a psychiatric misdiagnosis before their hEDS diagnosis. As depicted in Figure 2 and Table 2, 378 patients (88%) were told that they were fabricating their symptoms, 326 patients (76%) were labeled as attention-seeking, 286 patients (67%) were misdiagnosed with conversion disorder, 255 patients (60%) were told that it was “in their head”, and 16 patients (4%) were diagnosed with Munchausen syndrome by proxy or a factitious disorder. Statistical analyses by age group are illustrated in Figure 3, Figure 4, Figure 5, Figure 6, Figure 7 and Figure 8. A chi-square test between the age groups yielded a *p*-value of 0.423, a degree of freedom of 20, and a chi-square statistic of 20.568, as shown in Table 3.

## 4. Discussion

As illustrated in Figure 1 and Figure 2, patients with complex medical conditions such as hEDS are frequently misunderstood and misdiagnosed. Prior to receiving an accurate diagnosis of hEDS, 94.4% of patients were incorrectly identified as having psychiatric disorders by physicians who were not board-certified psychiatrists. The most common misdiagnosis involved patients being accused of fabricating symptoms and pain, while the least common was a factitious disorder, at a rate of 4%. Only 5.6% of patients avoided psychiatric misdiagnosis altogether. As shown in Figure 3, Figure 4, Figure 5, Figure 6, Figure 7 and Figure 8, higher misdiagnosis rates were observed in patients who were younger than 30 and those aged 51 to 60. A chi-square test revealed a *p*-value of 0.423 and failed to reject the null hypothesis, as depicted in Table 3. We found that there is no statistically significant association between the age group and the distribution of misdiagnosis categories at the conventional 5% significance level.

Considering that misdiagnoses may have occurred at various time points prior to their visit for hEDS evaluation, an analysis without this information may have inflated the findings or disproportionately affected patients in certain age groups or those with longer diagnostic delays. In future research, analysis with information about the timing of their psychiatric misdiagnosis will be helpful.

The criteria for misdiagnosis required one or more psychiatric diagnoses that were not secondary to an organic cause by non-psychiatric board-certified physicians and a clinical diagnosis of hEDS. Given that hEDS diagnoses frequently occur years after symptom onset, self-reported data related to psychiatric misdiagnoses may be subject to recall bias. The authors did not have access to the patients’ previous psychiatric diagnosis dates, and the record was based on self-reported data. Many of the patients received psychiatric evaluations that proved that they did not have a primary psychiatric diagnosis. However, these were also self-reported information by the patients, and the authors did not have access to their previous psychiatric evaluations. These limitations, coupled with a selection bias, given that all study participants were recruited from a single specialty center that attracts complex patients, all lead to a high misdiagnosis rate.

This article further investigates the causes and contributing factors of misdiagnosis of hEDS and strategies to mitigate misdiagnosis. The high misdiagnosis rate of hEDS as psychiatric disorders can be partly explained by biases and limited awareness among healthcare providers [22]. Cognitive biases, such as anchoring (relying heavily on initial impressions) and availability bias (favoring recent or memorable cases), significantly influence diagnostic errors [23]. Once patients are mislabeled with psychiatric diagnoses, anchoring bias can impede accurate reassessment. Limited awareness and the relatively low prevalence of hEDS make it difficult for healthcare professionals, including psychiatrists, to recognize them and differentiate psychiatric presentations due to organic conditions from primary psychiatric conditions [17,22]. Non-specific EDS symptoms that mimic other disorders further elevate misdiagnosis risks [17]. Additional systemic factors, including poor communication, inadequate diagnostic tools, and time constraints, may exacerbate these diagnostic errors [24].

Patients with EDS often have co-diagnoses of postural orthostatic tachycardia syndrome (POTS), MCAS, gastroparesis, and median arcuate ligament syndrome [25,26,27]. This nature of multiple co-diagnosis in EDS adds complexity to the correct diagnosis of EDS. This problem is not only limited to EDS and applies to other chronic pain conditions, including fibromyalgia, complex regional pain syndrome (CRPS), and chronic migraine [28,29,30]. Patients often face misunderstandings and dismissal by healthcare providers. Misdiagnoses as primary psychiatric disorders lead to inappropriate treatments, such as psychotherapy or anxiolytic medications, while their physical issues remain unaddressed. Delayed care and psychological distress from misdiagnosis can lead to unnecessary trust erosion and emotional harm.

The accurate diagnosis of chronic pain disorders benefits from a multidisciplinary approach, involving pain specialists, rheumatologists, neurologists, and qualified mental health professionals. Ensuring accuracy involves a thorough medical history and physical examination, structured diagnostic criteria such as DSM-5, validated screening tools (MINI International Neuropsychiatric Interview, Beck Anxiety Inventory), efforts to minimize cognitive biases, patient-centered care, enhanced diagnostic training, technological integration, and improved communication strategies [31,32,33,34]. Encouraging patients to ask questions and share their complete medical history can help clinicians gather comprehensive information [33]. Standardizing diagnostic protocols and promoting error reporting without punitive repercussions further mitigate misdiagnoses [34].

The authors acknowledge that although this project was determined not to be research involving human subjects as per the Brown University Human Research Protection Program, sensitive patient information was handled carefully to ensure confidentiality and minimize harm.

## 5. Conclusions

Misdiagnosing EDS as psychiatric illness profoundly impacts patients’ physical, emotional, and economic well-being. In our study, 94.4% of patients who were clinically diagnosed with hEDS were misdiagnosed with psychiatric illnesses by physicians who were not board-certified psychiatrists. The most common misdiagnosis involved accusations that patients were fabricating their pain. Addressing underlying causes and improving strategies to recognize EDS more accurately can substantially improve outcomes for patients who are affected by these complex disorders.

## Figures and Tables

**Table 1:** Screening questionnaires used in intake form.

Have You Ever Been Told That	Yes	No
Your condition is in your head?		
You are making it up?		
You have conversion disorder		
You are “attention-seeking”		
Accused of Munchausen by proxy or Munchausen		

**Table 2:** Prevalence of misdiagnosis as psychiatric illnesses by physicians who were not board-certified in psychiatry.

Total Number of Participants	429
Your condition is in your head?	255
You are making it up?	378
You have conversion disorder	286
You are “attention-seeking”	326
Accused of Munchausen by proxy or Munchausen	16
Never have been misdiagnosed	24

**Table 3:** Chi-square test of independence between 6 age groups.

Chi-Square Statistics (χ^2^)	Degrees of Freedom	*p*-Value
20.568	20	0.423

## Data Availability

The data that support the findings of this study are available from the corresponding author upon reasonable request.

## Abbreviation

The following abbreviations are used in this manuscript:

EDS	Ehlers–Danlos Syndrome
